# Effect of Nanoparticles on the Morphology, Thermal, and Electrical Properties of Low-Density Polyethylene after Thermal Aging

**DOI:** 10.3390/nano7100320

**Published:** 2017-10-12

**Authors:** Youyuan Wang, Can Wang, Zhanxi Zhang, Kun Xiao

**Affiliations:** State Key Laboratory of Power Transmission Equipment & System Security and New Technology, Chongqing University, Chongqing 400030, China; y.wang@cqu.edu.cn (Y.W.); zhx.zhang@cqu.edu.cn (Z.Z.); xiaokun@cqu.edu.cn (K.X.)

**Keywords:** LDPE nanocomposites, FTIR spectra, space charge, dielectric properties, thermal aging

## Abstract

This paper investigates the morphology, thermal, and electrical properties of LDPE (low-density polyethylene)-based nanocomposites after thermal aging. The FTIR (Fourier transform infrared spectroscopy) spectra results show that thermo-oxidative reactions occur in neat LDPE and LDPE/SiO_2_ nanocomposites when the aging time is 35 days and in LDPE/MgO nanocomposites when the aging time is 77 days. Specifically, LDPE/MgO nanocomposites delay the appearance of thermo-oxidative reactions, showing anti-thermal aging ability. Furthermore, nanocomposites present lower onset degradation temperature than neat LDPE, showing better thermal stabilization. With regard to the electrical properties, nanocomposites maintain the ability to suppress space charge accumulation after thermal aging. Additionally, in comparison with SiO_2_ nanocomposites and neat LDPE, the permittivity of LDPE/MgO nanocomposites changes slightly after thermal aging. It is concluded that LDPE/MgO nanocomposites have better insulation properties than neat LDPE after thermal aging, which may be caused by the interface introduced by the nanoparticles.

## 1. Introduction

Polyethylene is widely used for energy storage, electronic, packing, and insulation material due to its excellent physical and chemical properties [1,2,3,4]. However, with the development of technology, it is necessary to research materials with better mechanical, thermal, and insulation characteristics. Therefore, many researchers have used nanotubes and nanoparticles, such as ZnO, MgO, SiO_2_, TiO_2_, and SiC, to prepare polymer-based nanocomposites [5,6,7,8,9,10,11,12]. It has been proven that nanocomposites have higher elongation at break, dielectric breakdown strength, and volume resistivity and have the ability to restrict the growth of electrical tree and suppress the space charge accumulation; they are considered to be the next generation materials in many areas. According to these studies, it has been recognized that the interface introduced by nanoparticles dominates the properties of nanocomposites [13,14]. On this basis, some models, such as the multi-core model and space charge model have been promoted to describe the interface between nanoparticles and the polymer matrix, which can be used to successfully explain the experimental phenomenon of nanocomposites [15,16].

However, in many situations, the high temperature may continuously affect the polymer during operation. Namely, thermal aging may progressively change the structure and the electrical properties of polymer, finally leading to the failure of insulation. Therefore, it is necessary to research the effect of thermal aging on the electrical properties of polymers and the polymer-based nanocomposites. To date, many researchers have pointed out that thermal aging can accelerate the thermo-oxidative reactions in polymers, leading to the β-scission and, hence, producing a large amount of free radicals, which can destroy the structure, especially the amorphous structure of polymers [17,18]. It has also been proven that the change of structure during thermal aging can decrease the thermal stabilization, mechanical and electrical insulation properties, presenting lower onset degradation temperature, lower dielectric breakdown strength, higher dielectric loss, and so on [2,19,20,21,22,23,24]. Nevertheless, some researchers have pointed out that Cu/polyethylene composites and TiO_2_/polypropylene nanocomposites showed anti-aging ability to a certain degree [25,26]. Specifically, Cu particles and TiO_2_ nanoparticles increase the thermal stabilization and mechanical properties of the polymer matrix after thermal aging. On the contrary, it is interesting that some other studies provided the opposite observations in polyethylene-clay nanocomposites and polypropylene/CaCO_3_ nanocomposites after thermal aging [27,28]. The clay nanoparticles and unmodified CaCO_3_, as well as the modified CaCO_3_, accelerate the thermal degradation of the polymer matrix, owing to the consumption of antioxidants by these nanoparticles during thermal aging.

In summary, nanocomposites have been proven to be able to improve the thermal stabilization, mechanical and electrical insulation materials of the polyethylene matrix. However, considering the high temperature during the operation of polymers, the properties, especially the electrical properties, of nanocomposites during thermal aging are still unclear. In order to ascertain whether nanoparticles can protect the structure and the electrical properties of the polyethylene matrix after thermal aging, we used SiO_2_ and MgO nanoparticles to prepare nanocomposites and researched the morphology, FTIR spectra, thermal stabilization, space charges behavior, and dielectric properties of these samples after thermal aging.

## 2. Experiment

### 2.1. Materials

The base polymer used was an additive-free low-density polyethylene (LDPE) with the grade of 2426 H and a melt flow index 2.1–2.2 g/min and a density of 0.910–0.925 mg/cm^3^ provided by Lanzhou Petrochemical Company (Lan Zhou, China). The nanoparticles used were hydrophobic SiO_2_ and MgO with 50 nm particle size provided by Aladdin Industrial Corporation (Shanghai, China). 

### 2.2. Sample Preparation

Neat LDPE and nanoparticles were melt-blended in a twin-screw extruder at 423 K. Nanocomposites, with a concentration of 1 wt %, were press molded at 393 K and at a pressure of 10 MPa, to produce films with a thickness of about 150 μm. Samples were degassed in a vacuum drier for seven days at 353 K. To investigate the properties of samples after thermal aging, The LDPE and nanocomposite samples were heated continuously in a temperature-controlled oven at 363 K for 77 days. The properties of samples were tested when the aging time was 0, 14, 35, 56, and 77 days. Before test, the samples for the scanning electron microscopy (SEM) analysis were fractured in liquid nitrogen directly to achieve the section surface of thin films, washed with hydrogen peroxide and distilled water, then coated with gold, which is a highly conducting material to prevent local charging from the scanning electron beam [19,29]. Prior to dielectric analysis, the gold electrodes were deposited onto both surfaces of the specimens by sputtering. The sputtered electrodes were 15 mm in diameter. For the FTIR analysis and space charge measurement, the samples with the thickness of 150 μm can be used directly.

### 2.3. Scanning Electron Microscope Imaging

The sample section surface, fractured in liquid nitrogen, was observed by scanning electron microscopy (SEM, MERLIN Compact, Quantum Design, San Diego, CA, USA) in order to observe the morphology of nanocomposites after thermal aging. The extra high tension (EHT) and the magnification (Mag) are 2 kV and 5000×.

### 2.4. FTIR Analysis

FTIR was applied to evaluate the change of functional groups of the neat LDPE and nanocomposites after long-term thermal aging. The FTIR transmission spectra were obtained in the range of 400–4000 cm^−1^ by using Bruker ALPHA FTIR spectroscopy (Thermo Nicolet Corporation, Madison, WI, USA). The extent of thermal aging of LDPE and nanocomposites can be characterized by the carbonyl index, which was calculated by Equation (1) [30]:

(1) $$\text{Carbonyl}\;\text{index}=\frac{P_{c}}{P_{r}},$$

where *P_c_* is the absorption at 1720 cm^−1^ (the maximum of the carbonyl peak), *P_r_* is the absorption at 1464 cm^−1^ (–CH_2_ scissoring peak).

### 2.5. Thermogravimetric Analysis (TGA) 

Thermogravimetric analysis (TGA) was conducted in a Mettler Toledo TGA device (ShenZhen BEL Technology Co., ShenZhen, China) at a heating rate of 10 °C/min. Tests were run in nitrogen atmosphere in the temperature range from 30 to 600 °C.

### 2.6. Space Charge Measurments

The space charge measurements were carried out with a pulsed electro-acoustic (PEA) system (Texas Instruments, Dallas, TX, USA), the pulse width of which is 2–5 ns, pulse amplitude is 200 V and output voltage is 0–20 kV. All samples were measured at room temperature (25 ± 1 °C). In this paper, a 20 kV/mm DC (direct current) electrical field was applied for 1200 s and the space charge formation was confirmed with the polarization of 1200 s. The resolution of PEA device is about 1.2 × 10^−3^ C/m^3^ and the reproducibility of the space charge behavior was confirmed by repeating the experiments five times for each group.

### 2.7. Dielectric Properties

The dielectric properties of the materials were measured in the frequency domain from 10^−1^ Hz to 10^6^ Hz at room temperature (25 ± 1 °C) by using a Novocontrol ALPHA-A high-resolution dielectric analyzer (Novocontrol Technologies, Montabaur, Germany). The results are the average measurements of five different specimens for each sample and the error in the measurement is within 2%.

## 3. Results and Discussion

### 3.1. SEM Morphology

**Figure 1:**
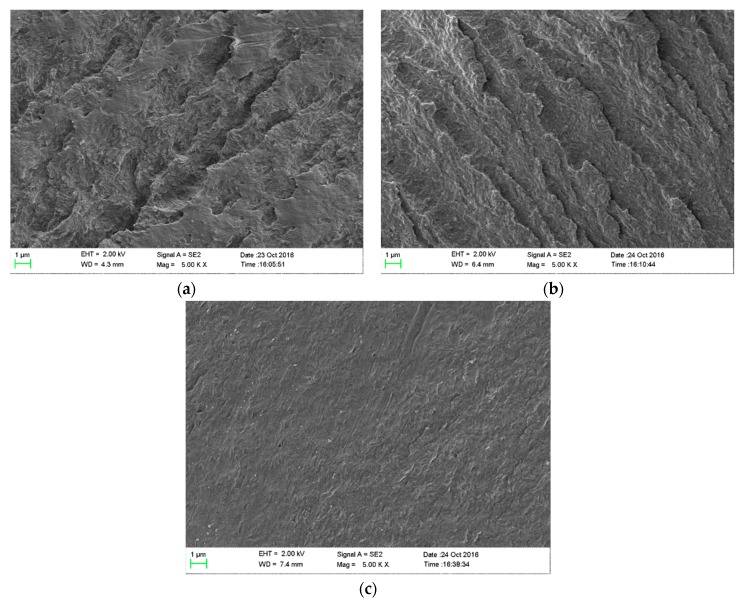
A sectional morphology graph of neat low-density polyethylene (LDPE) and 1 wt %-nanocomposites with an aging time of 77 days. (**a**) Scanning electron microscopy (SEM) image of neat LDPE; (**b**) SEM image of LDPE/SiO_2_ nanocomposites; (**c**) SEM image of LDPE/MgO nanocomposites.

The thermal aging can accelerate the β-scission of polymer chains and produce free radicals, leading to the destruction of the structure of polymers, which can be directly reflected by the morphology images. Therefore, we used SEM to observe the sectional surface of samples after thermal aging. The results are shown in Figure 1. After thermal aging, it is noted that gully structures are observed in neat LDPE and LDPE/SiO_2_ nanocomposites, representing the destruction of the structure of polymers during thermal aging. However, in the LDPE/MgO nanocomposites, it seems that the morphology is much smoother than that of neat LDPE and LDPE/SiO_2_ and no gully structures are observed in this kind of samples. Specifically, in comparison with SiO_2_ nanoparticles, MgO nanoparticles have the ability to protect the structure of the polyethylene matrix during thermal aging.

### 3.2. FTIR Spectra

**Figure 2:**
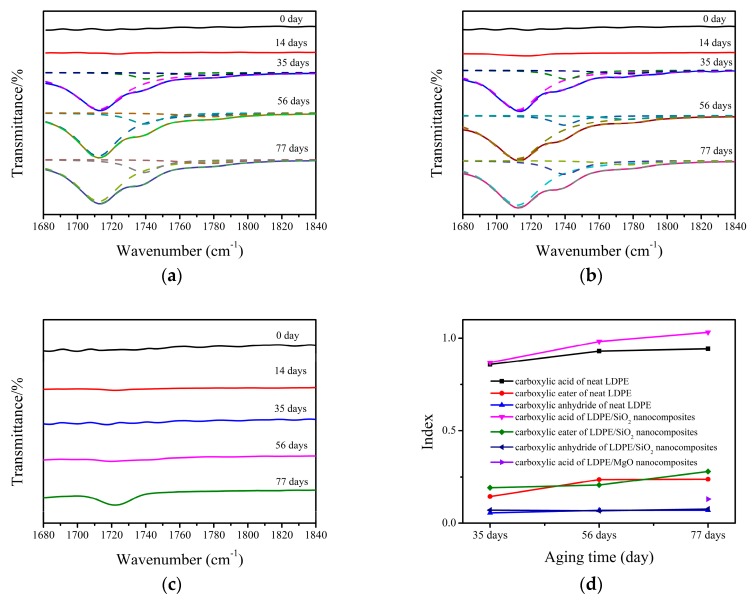
Fourier transform infrared spectroscopy (FTIR) spectra and carbonyl index of neat LDPE and 1 wt %-nanocomposites (**a**) FTIR spectra of neat LDPE, the dashed lines represent the fitting results; (**b**) FTIR spectra of LDPE/SiO_2_ nanocomposites, the dashed lines represent the fitting results; (**c**) FTIR spectra of LDPE/MgO nanocomposites; (**d**) Carbonyl index of all samples.

FTIR spectra are very sensitive to the change of molecular groups in polyethylene during thermal aging, which can be used to identify the new molecular groups and subsequently assess the thermooxidative degree of materials. As we know, the spectra domain from 1680 cm^−1^ to 1840 cm^−1^ represents the *v*_c=o_ vibration stretching peaks, which are the typical source of identifying thermooxidative reactions in the polyethylene matrix. In Figure 2, we show the FTIR spectra of neat LDPE and nanocomposites before and after thermal aging. It can be seen that the new vibration stretching peaks occurred at 1718, 1738, and 1780 cm^−1^, representing carboxylic acid, carboxylic ester, and carboxylic anhydride in neat LDPE when an aging time is 35 days. Specifically, the thermooxidative reactions happened in neat LDPE at this time. Similarly, the FTIR spectra of LDPE/SiO_2_ nanocomposites also show these new vibration stretching peaks when the aging time is 35 days. However, we can see that the FTIR spectra of LDPE/MgO nanocomposites show much smaller new vibration stretching peaks when the aging time is 77 days. It seems that, in comparison with LDPE/SiO_2_ nanocomposies, LDPE/MgO nanocomposites can delay the appearance of thermooxidative reactions in the polyethylene matrix, showing an anti-thermal aging ability.

In order to analyze the effect of thermal aging on the productions, we use Lorentz formula to fit the FTIR spectra and subsequently calculate the carbonyl index of three kinds of productions. The results are shown in Figure 2d. Since the *v*_c=o_ vibration stretching peaks of LDPE/MgO nanocomposites occurred when the aging time was 77 days only, the carbonyl index of these materials is a dot in this figure. According to the calculated results, it is noted that the carbonyl index of neat LDPE increases obviously from 56 days to 77 days and the results tend to stabilize when the aging time is 77 days. Specifically, the molecular group which could easily react with oxygen was almost consumed when the aging time was 77 days and hence the thermooxidative productions increase more slowly at this time. However, for the LDPE/SiO_2_ nanocomposites, the carbonyl indices of three kinds of productions increased continuously from 35 days to 77 days, which were also higher than those of neat LDPE. It seems that more molecular groups react with oxygen in LDPE/SiO_2_ nanocomposites compared with neat LDPE. In addition, LDPE/MgO nanocomposites show a much smaller carbonyl index when the aging time is 77 days. 

### 3.3. TGA Analysis

**Figure 3:**
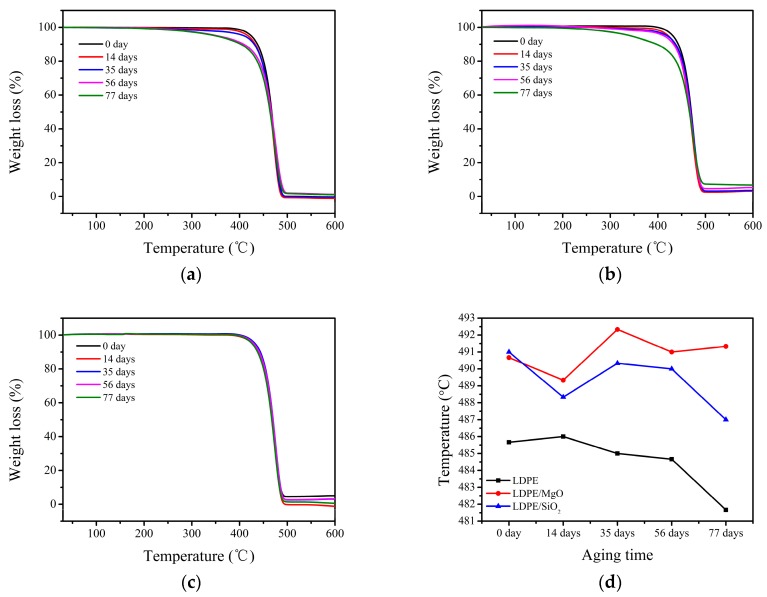
Thermogravimetric analysis (TGA) curves of neat LDPE and 1 wt %-nanocomposites with different aging times (**a**) TGA curve of neat LDPE; (**b**) TGA curve of LDPE/SiO_2_ nanocomposites; (**c**) TGA curve of LDPE/MgO nanocomposites; (**d**) temperature at 5% mass loss of all samples.

Figure 3 shows the thermograms of neat LDPE and nanocomposites with different aging times. The thermogram of neat LDPE shows that the onset degradation temperatures decrease obviously when the aging time is over 35 days, corresponding to the change of LDPE/SiO_2_ nanocomposites when the aging time is 77 days. However, it is different in LDPE/MgO nanocomposites, which remain almost unchanged during the whole thermal aging process. Additionally, Figure 3d gives the decomposition temperature at 5% mass loss, showing that the LDPE/MgO nanocomposites have the highest temperature after thermal aging. It is concluded that nanocomposites have better thermal stabilization than neat LDPE and LDPE/MgO nanocomposites have the best thermal stabilization. This may be caused by the interface between nanoparticles and the matrix, which can prevent the emission of small molecules during thermal aging.

### 3.4. Space Charge Distribution

The space charge will accumulate in polymer insulations, distorting the local electrical strength and hence leading to partial discharge. Therefore, locating and calculating the space charge accumulation in materials can help us to understand the weakness and assess the state of insulations.

**Figure 4:**
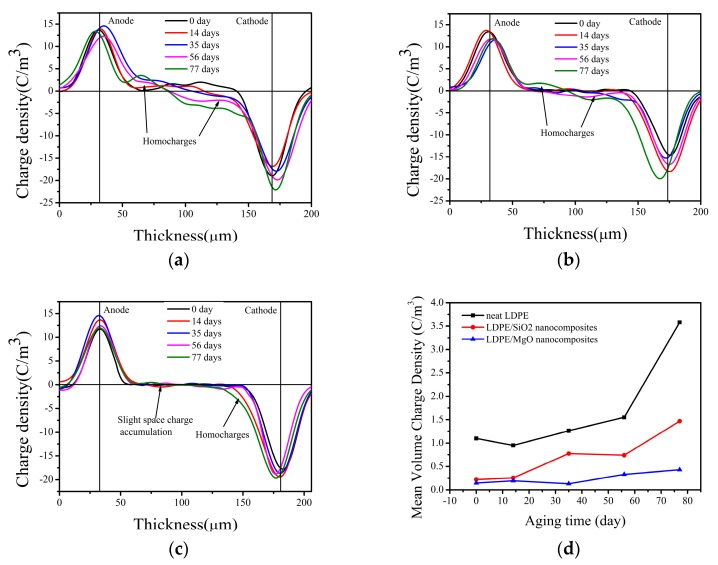
Space charge and mean volume charge density of neat LDPE and 1 wt %-nanocomposites (**a**) space charge curves of neat LDPE; (**b**) space charge curves of LDPE/SiO_2_ nanocomposites; (**c**) space charge curves of LDPE/MgO nanocomposites; (**d**) mean volume charge density.

Figure 4 shows the space charge distribution of neat LDPE and nanocomposites with different aging times. Each line in these figures represents the space charge behavior at different aging times when the polarization time is 1200 s. Obvious heterocharges are observed in neat LDPE before aging; however, both LDPE/SiO_2_ and LDPE/MgO nanocomposites show much fewer space charges than neat LDPE at this time, demonstrating the ability of nanoparticles to suppress space charge accumulation. After thermal aging, it can be seen that the heterocharges in neat LDPE change to homocharges and the amount of charges increase dramatically when the aging time is 77 days. Furthermore, the space charge distribution of LDPE/SiO_2_ nanocomposites also exhibit homocharges near electrodes. However, there are much fewer homocharges than neat LDPE. Subsequently, we can see that the LDPE/MgO nanocomposites show the best ability to suppress space charge after thermal aging with a slight space charge observed in samples.

In order to analyze the space charge amount in samples with the change of aging time, we calculate the mean volume charge density by using Formula (2) [31]:

(2) $$q\left(t;E_{p}\right)=\frac{1}{x_{1}-x_{0}}\int_{x_{0}}^{x_{1}}\left|q_{p}\left(x,t;E_{p}\right)\right|dx,$$

where *x*_0_ and *x*_1_ are the positions of the anode and cathode, respectively (induced charges at the electrodes are not taken into account), *t* is the depolarization time, *E_P_* is the polarization voltage, and $q_{P}(x,t;E_{P})$ is the charge density in the nanocomposites.

The results are shown in Figure 4d. In this figure, we can see that the charge density of neat LDPE and nanocomposites increases with the increasing of aging time and the neat LDPE has the highest charge density during the whole aging process. Specifically, nanocomposites have the ability to suppress space charge accumulation before and after thermal aging. Furthermore, before aging, LDPE/SiO_2_ nanocomposites have almost the same charge density as the LDPE/MgO nanocomposites. Thus, when the aging time is over 35 days, with the appearance of thermooxidative reactions, LDPE/SiO_2_ nanocomposites show a much higher charge density than LDPE/MgO nanocomposites. It is concluded that LDPE/MgO nanocomposites have the best ability to suppress space charge accumulation before and after thermal aging.

**Figure 5:**
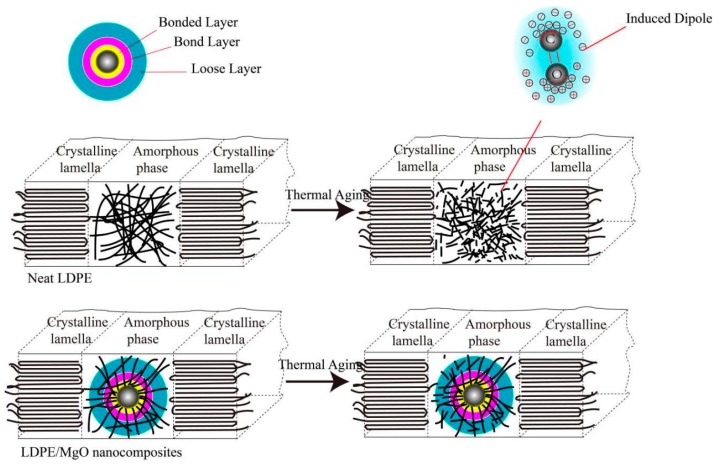
Illustration of neat LDPE and nanocomposites before and after thermal aging.

The space charge accumulation is caused by the charge traps distributed in the materials, which can trap carriers injected from electrodes. As we see from Figure 4a, the space charge behavior of LDPE shows obvious homocharges near electrodes after thermal aging. This may be caused by two reasons: (1) During thermal aging, the surfaces of the samples are exposed in air, leading to thermal-oxidative aging and hence destroy the structure of the surface. Specifically, the amount of defects distributed in this area will increase after thermal aging. These defects can act as charge traps and thus increase the homocharges near electrodes. (2) According to the research of Takada [32], the carbonyl groups (C=O) can act as induced dipoles under high voltage strength, whose electrical potential area can trap surrounding carriers. Specifically, these groups can be thought of as charge traps, which lead to homocharges after thermal aging. Figure 5 shows the change of structure of LDPE after thermal aging. The long polymer chains become smaller because of the β-scission of polymer chains. Nevertheless, the nanocomposites, especially the LDPE/MgO nanocomposites, exhibit much fewer space charges than neat LDPE after thermal aging. According to the research of Tanaka [16], the interface introduced by nanoparticles can be divided into three parts: bond layer, bonded layer, and loose layer. The bond layer and bonded layer can restrict polymer chains and hence increase the stabilization of the polymer matrix shown in Figure 5. The bond and bonded layer can restrict the movement of charge carriers and, hence, show much less space charge in nanocomposites after thermal aging.

### 3.5. Dielectric Properties

**Figure 6:**
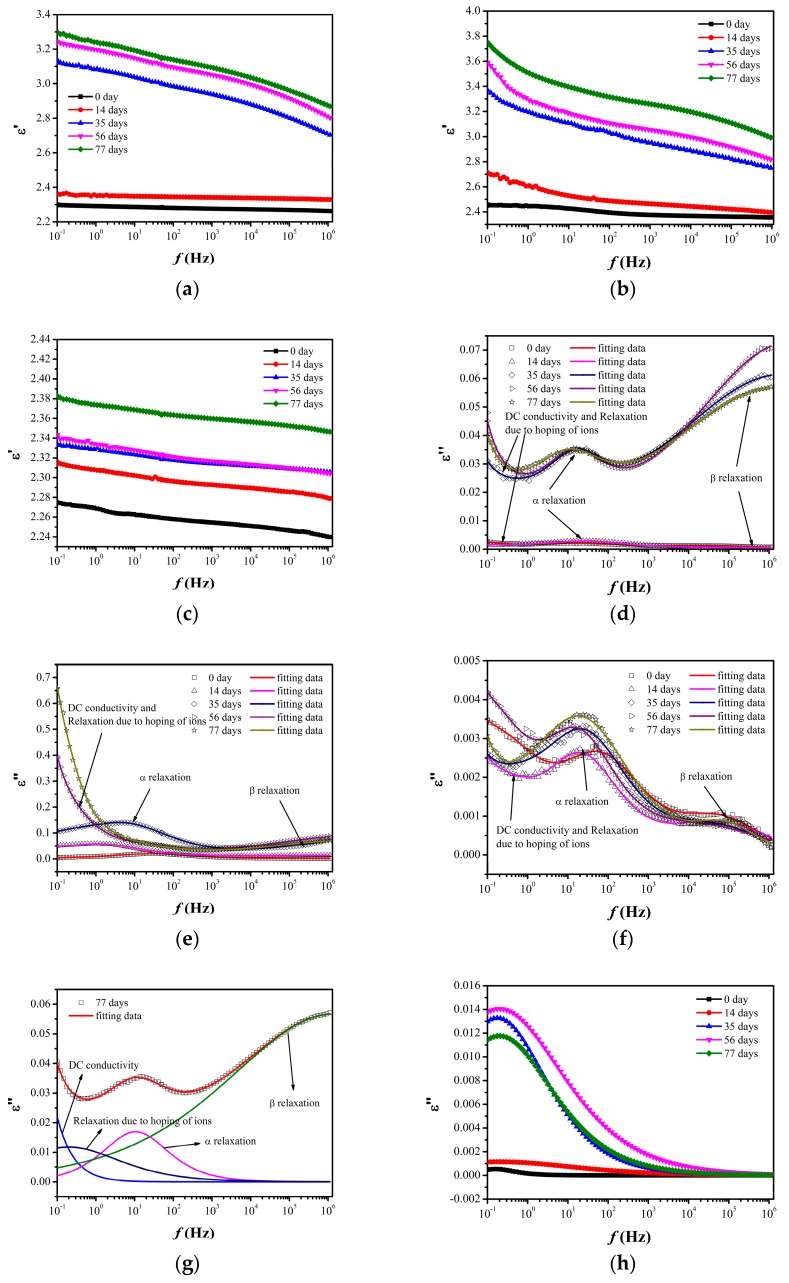

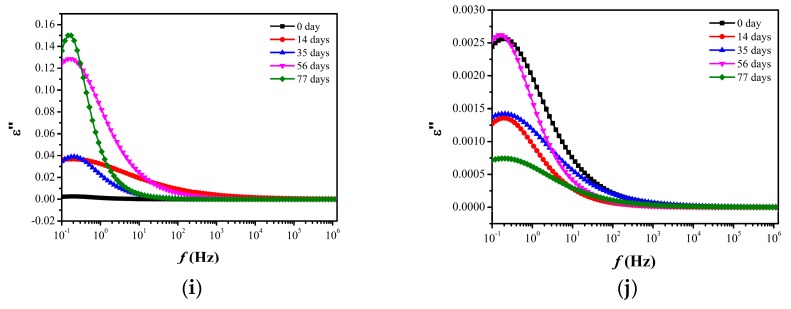
Permittivity of neat LDPE and nanocomposites before and after thermal aging (**a**) real permittivity of neat LDPE; (**b**) real permittivity of LDPE/SiO_2_ nanocomposites; (**c**) real permittivity of LDPE/MgO nanocomposites; (**d**) imaginary permittivity of neat LDPE; (**e**) imaginary permittivity of LDPE/SiO_2_ nanocomposites; (**f**) imaginary permittivity of LDPE/MgO nanocomposites; (**g**) fitting results of neat LDPE with the aging time of 77 days; (**h**) relaxation due to hopping of ions of neat LDPE; (**i**) relaxation due to hopping of ions of LDPE/SiO_2_; (**j**) relaxation due to hopping of ions of LDPE/MgO.

Figure 6 shows the real and imaginary permittivity of neat LDPE and nanocomposites before and after thermal aging, which are important factors for energy storage devices and insulation materials. As we see from the figure, the real permittivity of all samples increases with the increasing of aging time. Considering the result of the FTIR spectra, it is noted that the appearance of the dramatic increase in the real permittivity and the increase in slopes correspond to the appearance of thermo-oxidative reactions. Furthermore, in comparison with the neat LDPE, LDPE/SiO_2_ nanocomposites show much higher real permittivity, which reaches almost 3.8 at 10^−1^ Hz with the aging time of 77 days. However, the real permittivity of LDPE/MgO nanocomposites changes slightly after thermal aging. According to the imaginary permittivity of materials, it can be suggested that the imaginary permittivity contains three parts: relaxation due to the direct current (DC) conductivity and hopping of ions at low frequency, α relaxation and β relaxation at high frequency. In neat LDPE and LDPE/SiO_2_ nanocomposites, the value of the imaginary permittivity increases shapely and the relaxations become more obvious with the increasing aging time, especially when the thermo-oxidative reactions occurred in materials. In contrast, the imaginary permittivity of LDPE/MgO nanocomposites changes slightly and exhibits much smaller values than the other two materials, corresponding to the change of real permittivity of these materials during thermal aging.

In order to recognize the relaxations and DC conductivity of materials, we use the formula provided in [33] to fit the imaginary permittivity. More details about this formula can be found in [33]. 

The fitting results of neat LDPE with the aging time of 77 days and the relaxations due to hopping of ions are shown in Figure 6 and the DC conductivity of materials is shown in Table 1. It can be seen that with the increasing aging time, DC conductivity and the relaxations due to hopping of ions of neat LDPE and LDPE/SiO_2_ nanocomposites increase dramatically, especially with the appearance of thermooxidative reactions in materials. However, LDPE/MgO nanocomposites show much smaller DC conductivity and relaxation due to hopping of ions than the other two materials and these properties change slightly during the whole aging process, exhibiting the ability to protect DC conductivity and permittivity.

As we mentioned above, Takada [32] proved that the carbonyl groups (C=O) can be thought of as the charge traps. Furthermore, the calculation results from their research show that the contribution to trap depth by carbonyl groups (C=O) is about 0.45 eV, which means that the carbonyl groups (C=O) can be thought of as shallow charge traps. Specifically, the carriers can move across their electrical potential area by trap and de-trap. Therefore, with the increasing carbonyl groups (C=O) during thermal aging, the overlaps of the electrical potential area can provide the route for carriers, which can be thought of as the “quasi-conductivity area” and ultimately lead to the increase in DC conductivity. Finally, the neat LDPE and LDPE/SiO_2_ nanocomposites with obvious thermo-oxidative reactions show much higher DC conductivity than LDPE/MgO nanocomposites.

## 4. Conclusions

This paper investigated the effect of thermal aging on the morphology, thermal, and electrical properties of LDPE nanocomposites, revealing the anti-thermal aging ability of LDPE/MgO nanocomposites. Therefore, LDPE/MgO nanocomposites may be used as the insulation in high voltage equipment in the future. The conclusions are as follows:

(1) In comparison with SiO_2_ nanoparticles, MgO nanoparticles have the ability to protect the structure of the polyethylene matrix and delay the appearance of thermo-oxidative reactions during thermal aging. Furthermore, LDPE/MgO and LDPE/SiO_2_ nanocomposites have better thermal stabilization than neat LDPE.
(2) Nanocomposites have the ability to suppress space charge accumulation after thermal aging and the mean volume charge density of nanocomposites ranged from 0–1 C/m^3^ before and after thermal aging, which is much smaller than that of neat LDPE. It is suggested that the interface between nanoparticles and the polymer matrix may be the main reason for the suppression of space charge of nanocomposites.
(3) Neat LDPE and LDPE/SiO_2_ nanocomposites show much higher permittivity, which reach 3.3 and 3.8 with the aging time of 77 days, and more obvious relaxation peaks after thermal aging. The fitting results reveal that DC conductivity and the hopping of ions increase sharply after thermal aging. However, due to the anti-thermal aging ability of LDPE/MgO nanocomposites, the permittivity and DC conductivity change slightly during thermal aging, showing better insulation properties after thermal aging.

## Figures and Tables

**Table 1:** DC conductivity of materials from fitting data. LDPE: low-density polyethylene.

Aging Time	σ_dc_-Neat LDPE	σ_dc_-LDPE/SiO_2_	σ_dc_-LDPE/MgO
0 day	7.65 × 10^−15^	1.02 × 10^−16^	4.70 × 10^−15^
14 days	1.75 × 10^−15^	2.50 × 10^−14^	5.55 × 10^−15^
35 days	6.74 × 10^−14^	1.24 × 10^−13^	4.75 × 10^−15^
56 days	1.50 × 10^−13^	1.47 × 10^−12^	7.18 × 10^−15^
77 days	1.22 × 10^−13^	2.59 × 10^−12^	9.07 × 10^−15^
